# Deep vein thrombosis in a 14-year-old boy with a combination of May–Thurner syndrome, protein S deficiency and lupus anticoagulant: a case report

**DOI:** 10.1186/s13256-026-06028-5

**Published:** 2026-05-11

**Authors:** Vladislav Makeev, Ayesha Zeb, Sanjay Gupta

**Affiliations:** Department of Paediatrics, University Hospitals Birmingham NHS Foundation Trust, Birmingham, UK

**Keywords:** May–Thurner syndrome, Protein S deficiency, Lupus anticoagulant, Antiphospholipid syndrome, Deep vein thrombosis, Rivaroxaban, Warfarin, Case report

## Abstract

**Background:**

Here, we present the first reported case of a child presenting with unprovoked deep vein thrombosis (DVT) due to the unique combination of May–Thurner syndrome, protein S deficiency and lupus anticoagulant. Of interest to the clinician and scientific reader alike is that this one-of-a-kind interplay between mechanical obstruction and hypercoagulability caused a very extensive blood clot which extended from the left popliteal to the femoral and iliac veins. A 12-month course of warfarin with a target INR value of 2.5 was used to treat the DVT which would be of use to note for any future clinicians treating children presenting with extensive DVTs and multiple pro-thrombotic risk factors.

**Case presentation:**

A 14-year-old Asian (Han Chinese) boy presented to the children’s assessment unit with a 4-day history of left-sided groin pain radiating to the left knee. On examination his left thigh was swollen. Initial blood tests showed no clotting abnormalities and a normal platelet count. An ultrasound doppler of the lower limb veins was performed which showed an extensive thrombus involving the left popliteal, femoral, and iliac veins. An MRI of the abdomen and pelvis demonstrated that the left common iliac vein was narrowed by the left common iliac artery thereby confirming May–Thurner syndrome. He was started on enoxaparin bridging therapy to rivaroxaban initially. Further blood tests were positive for lupus anticoagulant. He was also found to be protein S deficient. After further discussions with haematological services a 12-month course of warfarin was indicated due to rivaroxaban being contraindicated in patients with antiphospholipid syndrome (APS). Our patient was suspected of having APS but to confirm, a second blood test had to be sent at least 12 weeks after the first. Warfarin was therefore started as we could not wait 12 weeks to confirm or refute a diagnosis of APS. The patient eventually tested negative for APS. After 12 months of treatment with warfarin, a repeat ultrasound doppler was performed which showed that the thrombus had almost entirely resolved with good flow in all the previously affected veins. He was subsequently started on a lower dose of rivaroxaban as maintenance therapy.

**Conclusions:**

Based on our literature search this is the first patient to be reported as having this unique combination of May–Thurner syndrome, protein S deficiency and testing positive for lupus anticoagulant. This case demonstrated that despite the child having such an extensive thrombus and presenting with multiple risk factors for thrombosis, that a 12-month course of warfarin with a target INR of 2.5 was sufficient in resolving his symptoms and facilitated the dissolution of the clot. This case also demonstrated the importance of considering APS as a risk factor for his clot as having this condition would contraindicate the use of DOACs. Testing negative for lupus anticoagulant on his repeat blood test, a reduced dose course of rivaroxaban (at 10 mg instead of 20 mg once daily) was indicated and was successful in ensuring that there was no recurrence of DVT with no reports of major or minor bleeds nor of any thromboembolic events occurring.

## Background

This presentation of unprovoked DVT was unique due to the extensive nature of the blood clot resulting from the one-of-a-kind combination of prothrombotic factors (May–Thurner syndrome, protein S deficiency and APS). May–Thurner syndrome is caused when the left iliac vein is compressed between the right common iliac artery and the lumbar vertebral body [1]. This condition is also referred to as Cockett syndrome or iliac vein compression syndrome. It is known to increase the risk of developing deep vein thrombosis. It classically presents as left leg lower limb swelling (with or without DVT) in a woman between the 2nd and 4th decade of her life [2]. It is uncommon for it to present in male paediatric patients [3].

Protein S deficiency can be caused by a nonsense mutation of the PROS1 gene and can also be caused by conditions affecting liver function (as protein S is produced by the liver) [4]. Protein S functions as an anticoagulant by inhibiting factor IXa and by serving as a cofactor for activated protein C (APC) and tissue factor pathway inhibitor (TFPI) [4]. Without it exerting the above anticoagulative effects, an individual would be susceptible to developing blood clots especially in the lower limbs [5].

The presence of lupus anticoagulant increases the risk of thrombosis as these IgG and IgM autoantibodies bind to phospholipid-binding proteins (such as prothrombin or beta1-glycoprotein I). Two blood tests positive for lupus anticoagulant taken at least 12 weeks apart and the presence of thrombosis such as DVT are key criteria for diagnosing APS [6].

## Case presentation

Deep vein thrombosis is a multifactorial disease requiring different aspects to be met from Virchow’s triad for thrombosis to occur. With interplay between hypercoagulable states and anatomical abnormalities we here present a case of a 14-year-old Asian (Han Chinese) boy developing a DVT. Our patient attended the children’s assessment unit with a 4-day history of left-sided groin pain, radiating to the left knee. There was no history of any trauma, insect bites or fevers. He had no significant past medical history. There was no known family history of clotting disorders or liver disease upon questioning both parents at the time of presentation. His left thigh and leg appeared more swollen compared to his right, otherwise his examination was normal. His bloods showed no abnormalities in terms of coagulation (INR was 1.2, with an APTT of 31.1 s and a prothrombin time of 14.3 s). His platelet count was within the normal range at 292/μL and his liver function tests were normal. His inflammatory markers were raised with a CRP of 49 mg/L and an ESR of 34 mm/hour, but he did not have leucocytosis present (white cell count was 9.35 × 10^9^/L). He was initially referred to the trauma and orthopaedic team to rule out osteomyelitis, septic arthritis, and reactive arthritis. On this basis, an MRI of the left femur was performed which did not identify any of the above but demonstrated oedema in the subcutaneous spaces and in the muscles with the presence of a thrombus in the left iliac and femoral veins.

**Fig. 1:**
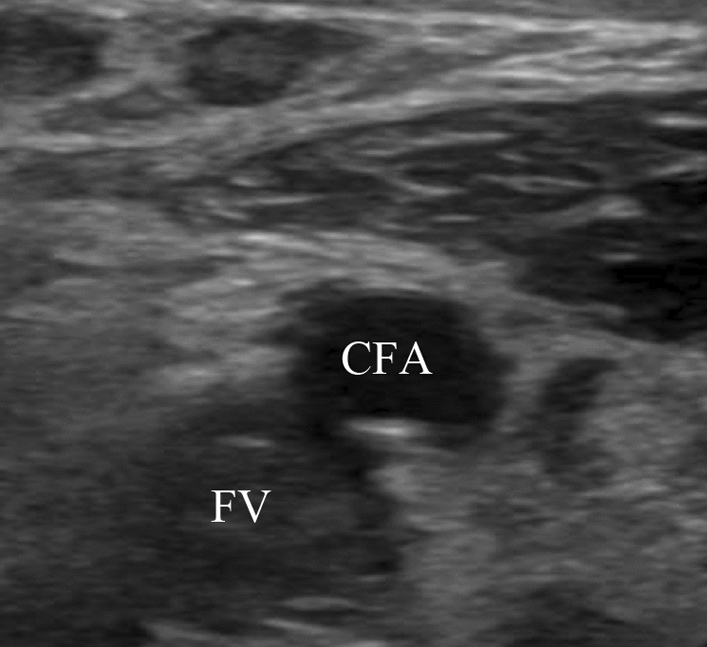
Ultrasound showing thrombus can be seen in left femoral vein

**Fig. 2:**
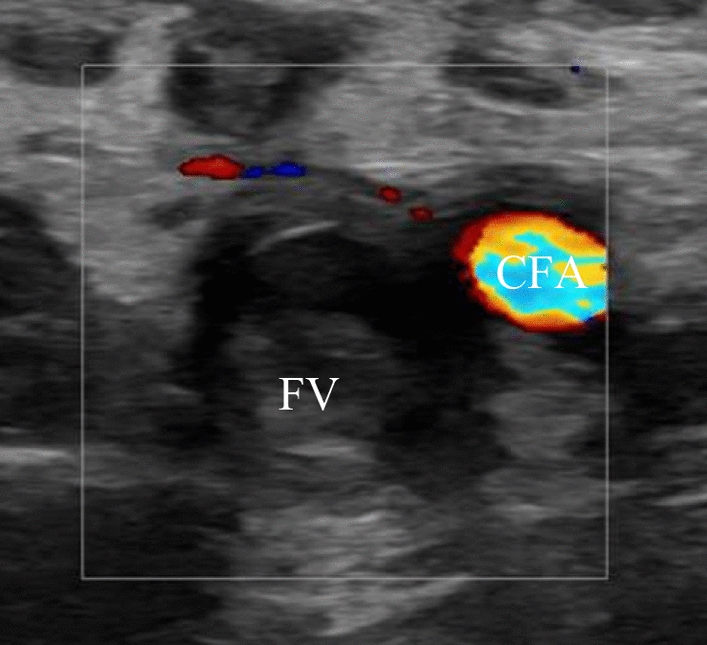
Doppler ultrasound showing no blood flow in the left femoral vein

**Fig. 3:**
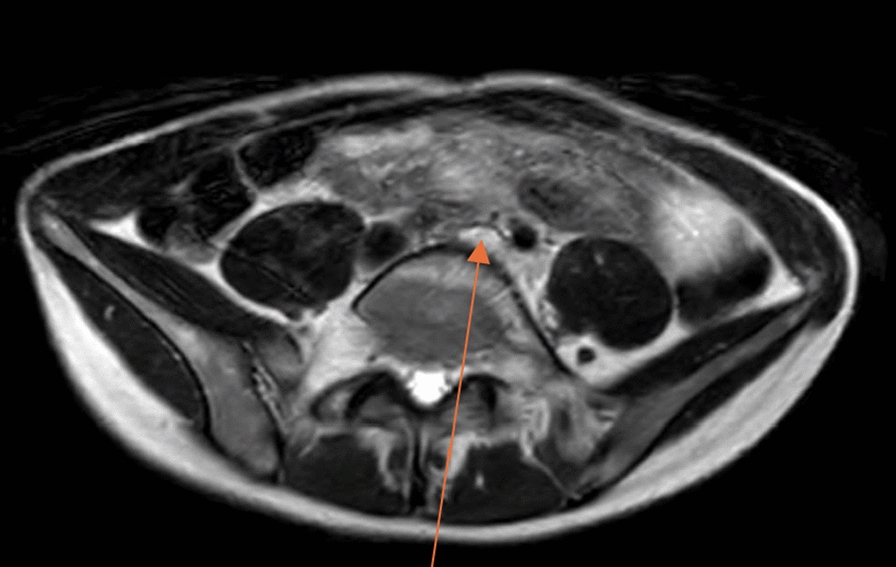
T2 weighted MRI with arrow pointing to thrombus in left common iliac vein where it is compressed

To identify the extent of the thrombus an ultrasound doppler of the lower limb veins was performed which confirmed the presence of a large thrombus occupying the left-sided calf, popliteal, femoral (Figs. 1, 2) and iliac veins. No blood flow was visualised on the doppler in these veins. The thrombus extended into the left side of the pelvis, but the inferior vena cava was clear at the level of the liver. His case was discussed with the regional haematological team who advised starting him on enoxaparin 60 mg twice daily for 5 days as bridging therapy to rivaroxaban 20 mg once daily initially. A further MRI was performed which demonstrated that the left common iliac vein was narrowed by the left common iliac artery indicating the presence of May–Thurner syndrome. The thrombus extended from this location (please see Fig. 3). Further bloods were requested including a coagulation screen. The patient’s protein S free antigen came back at 22% (normal range 60–124%) indicating protein S deficiency. Additionally, an LA-PTT test was performed which came back as 58 s (with a normal range of 29.2–49.3 s) which indicated the presence of lupus anticoagulant. After further re-discussion with the regional haematological team, a 12-month course of warfarin was opted for with a target INR range of 2.5 in case the patient had APS. Repeat blood tests, carried out 12 weeks after the initial blood test, demonstrated that the patient did not have APS as lupus anticoagulant was not raised. The patients relatives were also tested for lupus anticoagulant and protein S deficiency. Both the patient’s sisters and his mother tested positive for protein S deficiency. At 12 months of warfarin treatment a repeat doppler ultrasound demonstrated good flow in all the previously affected veins and only a very small remaining thrombus. As maintenance therapy, rivaroxaban was again started at a dose of 20 mg once daily which was then reduced to a 10 mg once daily regimen. A repeat doppler ultrasound was scheduled to continue to monitor his progress.

## Discussion

Virchow had hypothesised that there are three factors leading to the propensity of thrombosis to occur (stasis of blood flow, hypercoagulability, and endothelial injury) [7]. Likewise with our case we can see that it is not structural abnormality alone (May–Thurner syndrome) causing stasis of blood flow which has led to our child developing deep vein thrombosis. In addition to stasis of blood flow there was another factor at play: hypercoagulability. This was caused by protein S deficiency and lupus anticoagulant. Reflecting Virchow’s observation of coagulation often being multifactorial, Avila et al. observed that 60% of patients with deep vein thrombosis who had May–Thurner syndrome were also found to have a concomitant risk factor [8]. It can, therefore, be stipulated that May–Thurner syndrome predisposes to DVTs, but other factors (endothelial injury or hypercoagulability) have to be present for thrombosis to occur.

It is also interesting to note that May–Thurner syndrome was first referred to by Virchow in 1851 who remarked on the propensity of iliofemoral deep venous thrombosis to occur on the left side [7]. At the time he would not have been aware of the mechanism predisposing individuals to having thrombosis on the left side as opposed to the right (compression of the iliac vein by right common iliac artery and the lumbar vertebral body). The case we have presented reflects Virchow’s observations as our child too had deep vein thrombosis on the left side of his body.

What makes this case so unique is that it is the first reported case of a child having both protein S deficiency and lupus anticoagulant present who develops deep vein thrombosis in the presence of May–Thurner syndrome. The reduction in the anticoagulative and antithrombotic properties caused by a deficiency in protein S would have made him more susceptible to thrombosis. Both protein S deficiency and the presence of lupus anticoagulant were the main causes of hypercoagulability which, in the context of stasis of blood flow (caused by May–Thurner syndrome) led to thrombosis.

The positive family history (his mother and both sisters are protein S deficient) makes the diagnosis of a hereditary form of protein S deficiency most likely. PROS1 mutations are inherited in an autosomal fashion explaining why both his sisters and his mother have protein S deficiency [9]. The absence of any paternal family history of protein S deficiency makes it unlikely that the patient was homozygous for PROS1 mutations. Our patient having a homozygous form of protein S deficiency is made more unlikely given that individuals who develop protein S deficiency in a homozygous form often have severe clinical manifestations, often presenting in the neonatal period with purpura fulminans [10] which our patient did not have.

At the recommendation of the local haematological team, the patient was initially treated with a DOAC (rivaroxaban). This was mainly for two reasons: this oral medication is better tolerated in paediatric patients as it is easier to administer than parenteral medication like low molecular weight heparin (LMWH) and it is better tolerated as it requires less frequent monitoring compared to warfarin. It was decided that bridging therapy was required in the form of enoxaparin for 5 days prior to starting rivaroxaban. The reason cited by the regional haematological team were the British Society of Haematology (BSH) paediatric guidelines [11]. These guidelines refer to the EINSTEIN-Jr phase 3 trial and the DIVERSITY phase 2b/3 non-inferiority trial as evidence for their recommendation. It is these trials which used standard-of-care parenteral anticoagulant such as LMWH, unfractionated heparin (UFH) or fondaparinux as bridging therapy to a DOAC for 5–9 days and 5–21 days, respectively [12, 13]. It is interesting to note that this was done out of practicality as the participants in these studies had to be started on standard-of-care parenteral anticoagulation for a few days whilst awaiting consent to be gained from their parents to enrol them onto the trial and initiate DOAC treatment. It is interesting to consider whether it is necessary to commence paediatric patients on standard-of-care parenteral anticoagulation prior to starting DOACs since adult patients can be initiated immediately on DOACs without this bridging therapy. Nevertheless, our patient was started on enoxaparin based on BSH guidelines which recommend this practice. This treatment was all initiated before the results of the patient’s thrombophilia screen came back. This is very significant as the results of the thrombophilia screen were not expected and could have led to a diagnosis where the above treatment was contraindicated.

The patient’s thrombophilia screen showed that he was positive for lupus anticoagulant. This created an element of diagnostic uncertainty regarding whether the patient had APS or not as APS can be diagnosed by having thrombosis with two blood tests positive for antibodies associated with APS taken at least 12 weeks apart [6]. A diagnosis of APS would contraindicate the use of rivaroxaban (and other DOACs) because APS patients are at risk of higher rates of thromboembolic events as evidenced in trials such as TRAPS or that conducted by Ordi-Ros et al. [14, 15].

Due to the possibility of the patient having APS a different anticoagulant had to be found to treat the patient. The frequency of thromboembolic events in the TRAPS trial is cited as the reason why the BSH guidelines and the 2019 European Society of Cardiology (ESC) guidelines recommended against the use of DOACs in APS patients favouring the use of vitamin K antagonists (VKAs) such as warfarin instead. Our patient was, therefore, switched to warfarin and a repeat lupus anticoagulant blood test was requested. The second set of bloods demonstrated that he did not have APS.

After completing 12 months of treatment with warfarin and having a repeat doppler ultrasound which showed that the clot had almost entirely resolved, it was important to consider the future direction of anticoagulative therapy. The patient still had risk factors for thrombosis, and it is known that recurrent thrombosis is not an uncommon occurrence with patients presenting with any one of our patients risk factors. For example, venous thromboembolic events occurred in 55% of people with protein S deficiency and were recurrent in 77% of these people [9]. In addition, May–Thurner syndrome has a 1–5% risk of causing DVT [16]. All these factors are likely to have a synergistic effect in increasing the risk a recurrence of DVT in our patient. For this reason, it was not unreasonable to decide to continue some form of anticoagulative therapy. A decision was made to initiate treatment with rivaroxaban at a 20 mg once daily dose to be then stepped down to a 10 mg once daily dose. The lower dose was opted for as it was felt it was the safest option to opt for as some form of anticoagulative therapy needed to be given to prevent a recurrence of DVT whilst a lower dose would likely reduce the risk of side effects, particularly bleeding. The patient also felt rivaroxaban would be better tolerated by him as it required less monitoring with fewer blood tests.

Lastly, we are happy to report that the patient was treated successfully with him being able to participate unhindered at school in physical exercise in the form of PE lessons and after-school sports activities. He did not suffer any major or minor episodes of bleeding, nor did he suffer from any thromboembolic events. Having turned 16 he is awaiting to be transferred to adult care in whose hands the decision lies to decide whether to continue rivaroxaban as his life-long anticoagulative treatment or not.

## Conclusion

Here we have presented the first reported case a patient developing deep vein thrombosis having this combination of protein S deficiency, lupus anticoagulant and May–Thurner syndrome. This case demonstrates the importance of considering thrombosis as a cause of lower limb swelling, particularly of the left side in young adults. This case highlights that when investigating the causes of deep vein thrombosis it is not enough to simply identify a structural abnormality causing stasis of blood flow (such as May–Thurner syndrome) but to investigate further including performing a thrombophilia screen. If it was not for performing the thrombophilia screen, then we would not have been able to identify that the child had protein S deficiency and lupus anticoagulant. Identifying prothrombotic risk factors is important as it has implications when deciding the type and duration of anticoagulation therapy to use. In our case the presence of lupus anticoagulant meant that the initial treatment which the patient was started on (rivaroxaban) was potentially contraindicated due to a possible diagnosis of APS. This is because rivaroxaban was shown to increase the occurrence of thromboembolic events as evidenced in phase 3 trials such as TRAPS. Our case demonstrated that it is safe to opt to treat DVT patients who have a possible diagnosis APS with warfarin whilst awaiting a blood test to confirm or refute the diagnosis of APS. Our patient was successfully treated with a 12-month course of warfarin therapy with dissolution of the clot being confirmed on doppler ultrasound. A 10 mg once daily rivaroxaban course was opted for to prevent further thrombotic events. He has suffered no side effects from his anticoagulative medication including bleeding. Finally, we are pleased to report that the child has been able to enjoy all his regular school physical activities and has remained symptom free with no recurrence of DVT.

## Data Availability

Not applicable.

## Abbreviations

DVT: Deep vein thrombosis
APS: Antiphospholipid syndrome
MRI: Magnetic resonance imaging
APC: Activated protein C
TFPI: Tissue factor pathway inhibitor
LMWH: Low molecular weight heparin
UFH: Unfractionated heparin
SLE: Lupus erythematosus
APTT: Activated partial thromboplastin clotting time
LA-PTT: Lupus anticoagulant partial thromboplastin clotting time
DOAC: Direct oral anticoagulant
VKA: Vitamin K antagonist
